# A Neurotoxic *Ménage-à-trois*: Glutamate, Calcium, and Zinc in the Excitotoxic Cascade

**DOI:** 10.3389/fnmol.2020.600089

**Published:** 2020-11-26

**Authors:** Alberto Granzotto, Lorella M. T. Canzoniero, Stefano L. Sensi

**Affiliations:** ^1^Sue and Bill Gross Stem Cell Research Center, University of California, Irvine, Irvine, CA, United States; ^2^Center for Advanced Sciences and Technology (CAST), University “G. d’Annunzio” of Chieti−Pescara, Chieti, Italy; ^3^Department of Neuroscience, Imaging, and Clinical Sciences (DNISC), Laboratory of Molecular Neurology, University “G. d’Annunzio” of Chieti−Pescara, Chieti, Italy; ^4^Department of Science and Technology, University of Sannio, Benevento, Italy; ^5^Institute for Memory Impairments and Neurological Disorders, University of California, Irvine, Irvine, CA, United States

**Keywords:** Alzheimer’s disease, Amyotrophic lateral sclerosis, Huntington’s disease, Parkinson’s disease, mitochondria, reactive oxygen species, reactive nitrogen species, NADPH-diaphorase

## Abstract

Fifty years ago, the seminal work by John Olney provided the first evidence of the neurotoxic properties of the excitatory neurotransmitter glutamate. A process hereafter termed excitotoxicity. Since then, glutamate-driven neuronal death has been linked to several acute and chronic neurological conditions, like stroke, traumatic brain injury, Alzheimer’s, Parkinson’s, and Huntington’s diseases, and Amyotrophic Lateral Sclerosis. Mechanisms linked to the overactivation of glutamatergic receptors involve an aberrant cation influx, which produces the failure of the ionic neuronal milieu. In this context, zinc, the second most abundant metal ion in the brain, is a key but still somehow underappreciated player of the excitotoxic cascade. Zinc is an essential element for neuronal functioning, but when dysregulated acts as a potent neurotoxin. In this review, we discuss the ionic changes and downstream effects involved in the glutamate-driven neuronal loss, with a focus on the role exerted by zinc. Finally, we summarize our work on the fascinating distinct properties of NADPH-diaphorase neurons. This neuronal subpopulation is spared from excitotoxic insults and represents a powerful tool to understand mechanisms of resilience against excitotoxic processes.

## Introduction

Excitotoxicity is a form of neuronal death triggered by excessive and/or sustained exposure to the amino acid glutamate, the primary excitatory neurotransmitter in the brain. Evidence accumulated in the past four decades indicates that excitotoxicity is a critical contributor to the neuronal demise occurring upon acute and chronic neurological conditions, like stroke, Alzheimer’s disease (AD), Huntington’s disease (HD), Amyotrophic Lateral Sclerosis (ALS), and Parkinson’s disease (PD) (Mehta et al., 2013).

Although, 50 years have passed since the first description of glutamate’s neurotoxic activity (Olney, 1969), therapeutic strategies set at counteracting these processes have been only partially exploited. In that regard, the targeting of upstream mechanisms of glutamate-driven neurotoxicity has produced, in the late 80s, an early wave of enthusiasm and fueled a level of optimism that has not been corroborated in the following years. These approaches have been found promising in preclinical models (Lee et al., 1999) but failed in clinical trials (Lee et al., 1999; Ikonomidou and Turski, 2002; Chamorro et al., 2016; Choi, 2020). Only riluzole and memantine, two drugs that target glutamate-driven neuronal death, have been approved for the treatment of ALS and AD, respectively.

Although, most of the preclinical findings failed “the bench to bed” translation, this experimental evidence has significantly helped dissect the molecular underpinnings of excitotoxicity. These studies have also helped provide support for the excitotoxic cascade hypothesis (Zivin and Choi, 1991; Choi, 2020). The construct posits that excitotoxic neuronal death is primarily mediated by the glutamate-driven activation of N-methyl-D-aspartate receptors (NMDARs) and the subsequent toxic intraneuronal accumulation of calcium (Ca^2+^). The NMDAR-driven Ca^2+^ overload is, in fact, a mandatory step in the process as most of the downstream mechanisms of the cascade, like the generation of reactive oxygen species (ROS; of mitochondrial and non-mitochondrial origin), or reactive nitrogen species (RNS), the concurrent mitochondrial dysfunction, metabolic impairment, as well as the activation of necrotic/apoptotic pathways, are all Ca^2+^-dependent processes (Lee et al., 1999; Lai et al., 2014; Bano and Ankarcrona, 2018; Choi, 2020; Swanson and Wang, 2020). However, Ca^2+^ is not alone, and other cations find a way to participate in the death banquet. Zinc (Zn^2+^) is, for instance, a VIP guest.

In the review, we provide a brief overview of the role of Zn^2+^ in the brain and discuss its neurotoxic properties and how they intertwine with the excitotoxic cascade. Finally, we focus on the distinct features of the NADPH-diaphorase neurons, a subpopulation spared from excitotoxic insults offering an intriguing model to further our understanding of neuroprotective mechanisms.

## Zinc Homeostasis and Its Role in Brain Functions

After iron, Zn^2+^ is the most abundant metal ion in the brain. The cation can be found in either structural or labile forms (Sensi et al., 2009). Structural Zn^2+^ is tightly bound to proteins/peptides and acts as a critical component for proper protein folding or as the catalytic/co-catalytic element required for several enzymes (McCall et al., 2000).

Labile, free Zn^2+^ is either stored in the lumen of intracellular organelles, like synaptic “zinkergic” vesicles, mitochondria, lysosomes, the endoplasmic reticulum (ER), and the Golgi apparatus, or bound to metallothioneins (MTs), a class of metal-binding redox-sensitive proteins (Maret, 1994). Under physiological conditions, cytosolic Zn^2+^ concentrations are kept in a picomolar to a low nanomolar range (Outten and O’Halloran, 2001) through the carefully orchestrated activity of Zn^2+^ transporters (ZnTs), Zrt-, Irt-related proteins (ZIPs), Zn^2+^-stores and binding proteins (Sekler et al., 2007; Sensi et al., 2009).

Zn^2+^ within synaptic vesicles is released, along with glutamate, during excitatory neurotransmission (Sensi et al., 2009). Once released in the synaptic cleft, the cation shapes the post-synaptic glutamate responses by modulating the activity of glutamatergic receptors, like NMDARs and the α-amino-3-hydroxy-5-methyl-4-isoxazolepropionic acid receptors (AMPARs) (Paoletti et al., 1997; Kalappa et al., 2015). Zn^2+^ exerts an inhibitory effect on NMDARs by acting on a high (nanomolar) and a low (micromolar) affinity site that is located on the GluN2A and GluN2B subunits, respectively (Rachline et al., 2005). As for AMPARs, the cation has been proposed to exert an inhibitory activity by acting on the histidine residues of the receptor ligand-binding domain (Kalappa et al., 2015). However, direct structural evidence for this interaction is still missing. Of note, recent findings indicate that ZnT1-dependent cation extrusion in the proximity of synaptic NMDARs is required for the Zn^2+^-dependent inhibition of the receptor (Mellone et al., 2015; Krall et al., 2020). A novel post-synaptic mechanism that may put under a new light the cation’s modulating activities as the metal has been so far thought to only act via its presynaptic release. Extracellular Zn^2+^ is also implicated in the modulation of neurotrophic signaling as the cation is critical for the activity of the matrix metalloproteinases (MMPs), a class of enzymes involved in matrix remodeling and the maturation of the brain-derived neurotrophic factor (BDNF) from its precursor form proBDNF (Hwang et al., 2005), a process activated by Zn^2+^ supplementation and impaired by metal chelation (Corona et al., 2010; Frazzini et al., 2018). The proBDNF/BDNF balance is critical for neuronal functioning as the two peptides exert opposite effects. BDNF affects long-term memory processes and neuronal survival. On the contrary, proBDNF inhibits GABAergic neurotransmission (Riffault et al., 2014), facilitates long-term depression (Woo et al., 2005), and activates neuronal death-related pathways (Teng et al., 2005; Mizui et al., 2016). Zn^2+^ has also been shown to activate the BDNF receptor TrkB directly. This process, called transactivation, is triggered by synaptically-released Zn^2+^ and/or ROS-driven intracellular Zn^2+^ elevations (Huang et al., 2008; Huang and McNamara, 2012). However, the mechanisms described “*in vitro*” settings do not entirely translate in “*in vivo*” conditions as, surprisingly, TrkB activation has been found to be unaffected in transgenic mice devoid of vesicular Zn^2+^ (Helgager et al., 2014).

Unlike what is known about vesicular Zn^2+^, the intracellular labile pools’ physiological significance has been only partially unraveled. Along with its role as a metal reservoir, compelling evidence indicates that releasable Zn^2+^ can affect mitochondria and lysosomal functioning and, in close analogy with Ca^2+^, act as a signaling molecule (Yamasaki et al., 2007).

Most importantly, like Ca^2+^, when dysregulated, Zn^2+^ can turn into a potent neurotoxin (Sensi et al., 2009).

## Zinc as Neurotoxin

The contribution of Zn^2+^ in neurodegenerative processes has been extensively investigated. In conditions characterized by the overactivation of excitatory signaling, synaptically released Zn^2+^ can flux into post-synaptic neurons through Zn^2+^ permeable channels (Sensi et al., 2009). Zn^2+^ entry occurs primarily through voltage-gated Ca^2+^ channels (VGCCs) and Ca^2+^ permeable AMPARs (CP-AMPARs) (Sensi et al., 1997, 1999b; McDonald et al., 1998; Colvin et al., 2000; Sheline et al., 2002). AMPAR permeability to Ca^2+^ and Zn^2+^ ions is restricted to certain neuronal populations or occurs upon disease associated challenges. The distinct expression pattern and the high permeability to Ca^2+^ and Zn^2+^ contribute to the unique role of CP-AMPAR in selective neurodegeneration (see Weiss, 2011 for an extensive review on the topic). NMDARs are poorly permeable to Zn^2+^ ions (Sensi et al., 1997).

Additional routes of entry are the Na^+^-Zn^2+^ exchanger and transient receptor potential channels (TRP); however, their contribution to the cation’s toxic accumulation is still mostly unexplored (Bouron and Oberwinkler, 2014). The exact amount of Zn^2+^ released from presynaptic terminals and the degree of its contribution to cation accumulation in the post-synaptic neurons are also not completely clear and have been matters of lively debates (Kay and Toth, 2008; Vergnano et al., 2014).

Zn^2+^ released from intracellular pools also participates in the cation’s cytosolic build-up (McCord and Aizenman, 2014). In this regard, MTs are a significant source of intracellular Zn^2+^ (Maret, 1994; Aizenman et al., 2000). MTs mobilize a large amount of Zn^2+^ (ranging 10–100 nM) in response to Ca^2+^-driven generation of ROS/RNS as well as in conditions of mild acidosis, a combination found in excitotoxic settings and several neurodegenerative conditions. The critical role played by Zn^2+^ released from MTs in the production of neuronal death is supported by the ability of oxidative agents [i.e., 2,2′-dithiodipyridine (DTDP) or N-ethylmaleimide (NEM)] to trigger widespread apoptotic neuronal death (Aizenman et al., 2000; Gibon et al., 2010). This process is mostly reduced by Zn^2+^chelators and independent of Ca^2+^ load. Intraneuronal Zn^2+^ rises are not the final step of the toxic cascade but are critical to trigger mitochondrial and lysosomal dysfunction, as well as the activation of neurotoxic pathways in the cytosol (Sensi et al., 2009; Ji et al., 2019; Koh et al., 2019).

Mitochondria are a primary target of intracellular Zn^2+^ as the cation accumulates in the organelles thanks to their steep electrochemical gradient (Δp). Once sequestered, Zn^2+^, along with Ca^2+^, contributes to Δp loss and promotes ROS generation (Sensi et al., 1999a; Ji and Weiss, 2018). Zn^2+^ mobilization is an essential prerequisite to trigger irreversible mitochondrial dysfunction as the cation, by acting in close synergy with Ca^2+^ damaging effects, promote the full demise of the organelles and, eventually, cell death (Jiang et al., 2001; Granzotto and Sensi, 2015). Within mitochondria, Zn^2+^ acts by inhibiting complexes of the electron transport chain (ETC) and α-ketoglutarate dehydrogenase (αKGDH) of the Krebs cycle, thereby promoting aberrant ROS production and metabolic failure (Sensi et al., 2009; Ji et al., 2019). Zn^2+^ interactions with αKGDH and the matrix-facing complexes of the ETC support the presence of the cation in the mitochondrial matrix. Moreover, recent findings indicate that mitochondrial Zn^2+^ uptake through the activation of the mitochondrial Ca^2+^ uniporter (MCU) participates in producing the neuronal death found in preclinical models of brain ischemia (Ji et al., 2019, 2020). Zn^2+^ also triggers the permeabilization of the mitochondrial membrane through the activation of the mitochondrial permeability transition pore (MPTP; a key promoter of cell death; Bernardi et al., 2015), thereby generating the release/production of pro-apoptotic factors [like cytochrome *c*, apoptosis-inducing factor (AIF), and ΔN-Bcl-X_*L*_] (Jiang et al., 2001; Bossy-Wetzel et al., 2004; Bonanni et al., 2006; Ji et al., 2019).

In addition, Zn^2+^ elevations target lysosomes (Koh et al., 2019). Lysosomal Zn^2+^ rises, coupled with the accumulation of lipid peroxidation byproducts (4-hydroxinonenal), are instrumental for organelle membrane permeabilization (LMP). LMP results in cation release in the cytosol, along with the activation of lysosomal degrading enzymes. These events are critical for neuronal and astrocyte death when exposed to oxidative challenges (Lee and Koh, 2010; Koh et al., 2019).

Zn^2+^ also affects many cytosolic pathways to promote demise in the CNS cells, including activation of apoptotic/necrotic pathways, modulation of plasma membrane channels, depletion of metabolic substrates, and the induction of cytosolic oxidative enzymes. In neurons and astrocytes, the metal contributes to NADPH oxidase activation, resulting in aberrant O_2_^–^ generation (Noh and Koh, 2000; Brennan et al., 2009; Swanson and Wang, 2020). Similarly, Zn^2+^ activates the neuronal isoform of the nitric oxide synthase (nNOS), thereby promoting increased production of nitric oxide (NO) (Kim and Koh, 2002). These two pathways converge in a process in which O_2_^–^ + NO generate ONOO^–^ (peroxynitrite), a potently neurotoxic RNS (Bossy-Wetzel et al., 2004). Of note, the Zn^2+^-driven ROS/RNS production promotes further metal release from intracellular redox-sensitive stores (like MTs), thereby exacerbating a vicious feed-forward loop of cation dyshomeostasis (Corona et al., 2011; Slepchenko et al., 2017). At the cytosolic level, Zn^2+^ promotes NAD^+^ depletion, thereby resulting in glyceraldehyde-3-phosphate dehydrogenase (GAPDH, a critical enzyme in the glycolytic pathway) inhibition, ATP breakdown, and eventually energetic neuronal failures (Sheline et al., 2000). This NAD^+^ depletion may critically impinge on mechanisms that are relevant to aging (Lautrup et al., 2019). Zn^2+^ also targets and promotes activation of PARP (Kim and Koh, 2002), cyclin-dependent kinase 5 (Cdk5; Tuo et al., 2018), and AMPK (Kim et al., 2020), three molecules involved in cell death pathways.

Finally, Zn^2+^ mobilization, by contributing to the activation of the CamKII/p38/syntaxin/calcineurin axis, promotes outward potassium (K^+^) currents, a critical step in the production of neuronal apoptosis (Yu et al., 1997; McCord and Aizenman, 2013; Shah and Aizenman, 2014; Aizenman et al., 2020).

## The Resilience of nNOS (+) Neurons: a Model to Investigate Excitotoxic Mechanisms

Intriguingly, some neuronal subpopulations are mostly insensitive to excitotoxicity. The phenomenon is present in oculomotor neurons, Onuf’s nucleus neurons, and NADPH-diaphorase neurons (Koh et al., 1986; Brockington et al., 2013). NADPH-diaphorase neurons are a subset of medium-sized aspiny interneurons that are largely spared following excitotoxic hits (Koh et al., 1986; Koh and Choi, 1988; Uemura et al., 1990; Weiss et al., 1994; Granzotto and Sensi, 2015). The subpopulation is characterized by the overexpression of nNOS [also known as NOS1; hereafter termed nNOS (+) neurons (Dawson et al., 1991; Hope et al., 1991)]. nNOS (+) neurons are present with a relatively more significant percentage in the striatum but are also expressed in good numbers in the hippocampus and the cerebral cortex. The subpopulation encompasses various cellular subtypes characterized by distinct morphological, transcriptomic, and functional features (Tricoire and Vitalis, 2012). Early studies have shown that these neurons survive instead of the widespread neuronal loss documented by brain autopsy of AD, HD, and PD patients, three conditions characterized by a robust glutamatergic overdrive (Ferrante et al., 1985; Graveland et al., 1985; Mufson and Brandabur, 1994).

Our group has recently exploited this neuronal subpopulation’s unique features to evaluate the mechanisms that promote resilience to excitotoxicity. Employing an array of single-cell imaging and biochemical approaches, we have demonstrated that nNOS (+) neurons fail to generate ROS in response to excitotoxic stimuli (Canzoniero et al., 2013; Granzotto and Sensi, 2015), a critical step that contributes to their resilience and enhanced survival upon glutamate-driven neurodegeneration.

The investigation of these processes has indicated an intriguing scenario in which the ROS-dependent release of intracellular Zn^2+^ acts as a critical intermediate step of the excitotoxic process (Granzotto and Sensi, 2015). Thus, experimental data support the notion that Zn^2+^ participates, with glutamate and Ca^2+^, in a neurotoxic *ménage-à-trois*.

Overactivation of NMDARs is the first mandatory step in the excitotoxic cascade; compelling evidence indicates that the receptor triggers the activation of early signaling pathways involving PSD95 and nNOS recruitment as well as aberrant Ca^2+^-driven induction of nNOS (Szydlowska and Tymianski, 2010; Fricker et al., 2018; Wu and Tymianski, 2018). Disruption of the NMDAR/PSD95/nNOS axis prevents excitotoxic damage in *in vitro* and *in vivo* preclinical models of cerebral ischemia (Aarts et al., 2002). Functional, transcriptomic, and biochemical analysis, however, indicate that nNOS (+) neurons express fully operational NMDARs that do not differ from the ones present in the general population of nNOS (−) neurons (Price et al., 1993; Landwehrmeyer et al., 1995; Canzoniero et al., 2013; Granzotto and Sensi, 2015; Granzotto and Sensi, 2015, observations). Interestingly, additional studies have also indicated that nNOS (+) neurons are positive to cobalt staining, a maneuver employed to identify CP-AMPARs, thereby suggesting that these cells possess a significant number of these glutamate receptor subtypes (Weiss et al., 1994). This set of findings supports the notion that NMDAR-driven Ca^2+^ overload and nNOS activation are necessary but not sufficient steps for the initiation and development of the excitotoxic cascade. Additional downstream processes are required, and Zn^2+^ participates in these mechanisms with a leading role.

### Mitochondria, the Final Common Pathway

Early studies indicated that mitochondria are critical hubs for the development of the excitotoxic cascade (Ankarcrona et al., 1995). The organelles participate in the clearance of NMDAR-driven cytosolic Ca^2+^ raises and are instrumental for the activation of apoptotic and necrotic processes (Ankarcrona et al., 1995; Schinder et al., 1996). Mitochondrial Ca^2+^ overload results in organelle dysfunction, aberrant ROS generation, and, ultimately, neuronal loss (Dugan et al., 1995; Stout et al., 1998; Duchen, 2012; Rizzuto et al., 2012).

**FIGURE 1:**
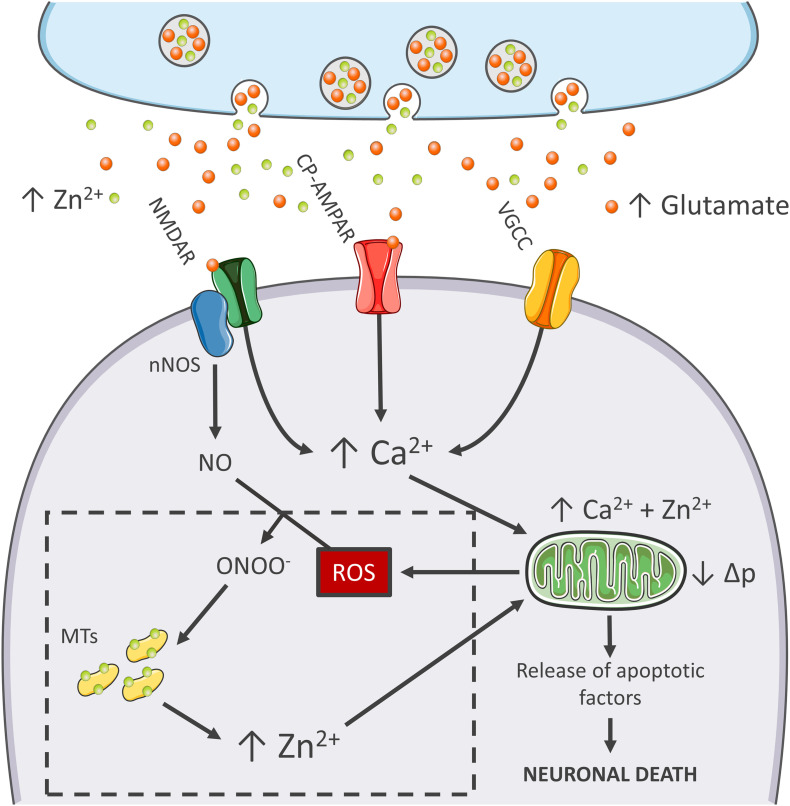
Zn^2+^ in the excitotoxic cascade. Aberrant release of glutamate from presynaptic terminals triggers NMDAR activation, which, in turn, promotes Ca^2+^ entry and generation of RNS and ROS of mitochondrial and extramitochondrial origin. The surge of ROS and RNS is required for intraneuronal Zn^2+^ mobilization from metallothioneins (MTs; Zn^2+^ buffering redox-sensitive proteins prone to release Zn^2+^ following oxidative stimuli). Intraneuronal Zn^2+^ rises target mitochondria and, along with Ca^2+^, contribute to the organelle impairment. Dysfunctional mitochondria fail to cope with Ca^2+^ clearance and further exacerbate Ca^2+^ dysregulation and ROS production. The lack of ROS generation in nNOS (+) neurons is a critical point of divergence in the excitotoxic cascade. By missing the injurious interaction between ROS and RNS, the subpopulation fails to mobilize neurotoxic Zn^2+^, prevents mitochondrial failure, and eventually neuronal death (dashed line box). In the general population of nNOS (−) neurons, pharmacological Zn^2+^ chelation prevents the full development of the excitotoxic cascade and mimics nNOS (+) cells’ behavior. These findings lend support to the idea that intraneuronal Zn^2+^ release is a critical regulator of excitotoxicity.

Mitochondria of nNOS (+) cells are insensitive to excitotoxicity and have emerged as a critical switch to turn off the injurious process (Canzoniero et al., 2013; Granzotto and Sensi, 2015; Figure 1). Although, mitochondria of these neurons take up large amounts of Ca^2+^, the organelles respond with minimal Δp losses and negligible generation of ROS (Canzoniero et al., 2013; Granzotto and Sensi, 2015). Early studies have shown that, to counteract the detrimental effects linked to peroxynitrite generation, nNOS (+) neurons express high levels of SOD2, the ROS quenching enzyme that is strategically localized inside of mitochondria (Gonzalez-Zulueta et al., 1998). Therefore, it is conceivable that this constitutive overexpression of SOD2 makes the subpopulation better equipped to cope with the oxidative surge produced by the excitotoxic challenges.

The idea that mitochondrial dysfunction and oxidative stress are prerequisites for NMDA-driven neuronal loss is in line with the “source-specific” hypothesis of excitotoxicity. The construct posits that the neurotoxic cascade depends on the route of Ca^2+^ entry, mainly NMDARs, rather than the magnitude of cation load (Wu and Tymianski, 2018). In agreement with this view, abundant Ca^2+^ entry through VGCCs, a maneuver devoid of neurotoxic effects, fails to trigger ROS and Δp changes (Table 1). This phenomenon shows great analogies with the effects of Ca^2+^ rises observed in nNOS (+) neurons following NMDAR activation (Granzotto and Sensi, 2015). Although, NMDAR and VGCC activation produces large Ca^2+^ rises, differences can be found when dissecting the temporal progression of the two stimuli. Unlike VGCC-driven Ca^2+^ entry, NMDAR overactivation promotes a prolonged and sustained build-up of Ca^2+^, a phenomenon likely due to impaired cation handling. Conceivably, the NMDAR-driven generation of RNS and ROS can severely affect the mitochondrial Ca^2+^ buffering as well as the defective extrusion of the cation.

**TABLE 1:** The functional hallmarks of excitotoxicity.

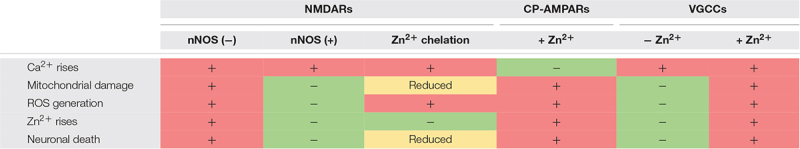

*The table compares the excitotoxic cascade-related changes occurring in neurons upon NMDAR activation in nNOS (−) and nNOS (+) neurons as well as in neurons in which Zn^2+^ mobilization is halted with a metal chelator (i.e., TPEN). The table also compares changes produced by neuronal depolarization, a maneuver that triggers massive Ca^2+^ entry through VGCC but is devoid of neurotoxic effects, and CP-AMPAR activation. Note that, unlike Ca^2+^, Zn^2+^ influx through VGCC mimics the excitotoxic cascade-related changes. In addition, the activation of CP-AMPAR in the presence of extracellular Zn^2+^ shows greater and long-lasting damaging effects when compared to experiments performed in the presence of physiological Ca^2+^ levels (Sensi et al., 1999b). Red, yellow, and green boxes indicate a significant change, a moderate change, and no change, respectively, in the parameters listed in the first column.*

RNS/ROS can *per se* contribute to mitochondrial damage (Murphy, 2009). However, an alternative angle is offered by the mitotoxic properties of Zn^2+^. The cation represents a critical point of convergence between Ca^2+^, ROS, and mitochondrial failure. By missing ROS generation, nNOS (+) neurons fail to mobilize intracellular Zn^2+^ upon NMDAR overactivation (Granzotto and Sensi, 2015). Chelation experiments support the hypothesis that NMDAR-triggered Zn^2+^ rises are required for the full development of the excitotoxic cascade. In that respect, chelation prevents Zn^2+^ rises without affecting the upstream mechanisms of the cascade (i.e., Ca^2+^ entry or the Ca^2+^-driven generation of RNS/ROS). In nNOS (−) neurons, blockade of excitotoxic-driven Zn^2+^ elevations results in reduced mitochondrial dysfunction and improved intracellular Ca^2+^ cycling; two functional changes that closely match the ones observed in the nNOS (+) subpopulation (Granzotto and Sensi, 2015) and provide neuroprotection in excitotoxic settings (Wang and Thayer, 2002). On the contrary, nNOS (+) neurons are extremely vulnerable to pharmacological maneuvers that promote Zn^2+^ elevations, thereby suggesting that the damaging effects of the cation can override the protective machinery of nNOS (+) neurons (Granzotto and Sensi, 2015, and unpublished observations).

These results are in line with several studies showing that Zn^2+^ chelation is highly neuroprotective as the maneuver prevents mitochondrial failure, irreversible dysregulation of Ca^2+^ homeostasis, and eventually neuronal demise (Jiang et al., 2001; Bossy-Wetzel et al., 2004; Medvedeva et al., 2009; Vander Jagt et al., 2009; Clausen et al., 2013; Medvedeva and Weiss, 2014; Ji and Weiss, 2018; Ji et al., 2020; Table 1).

The peculiar milieu offered by nNOS (+) neurons replicates these mechanisms in a naïve, patho-physiologically relevant setting and allows inference on the central role played by Zn^2+^ in the excitotoxic process (Figure 1). Zn^2+^ is, therefore, not an accomplice or an amplifier of Ca^2+^-driven toxicity but rather the downstream executioner. Zn^2+^ actively promotes mitochondrial dysfunction, Ca^2+^ dysregulation and, eventually, neuronal death.

## Revising the Role of Zinc in CNS Disorders

The nNOS (+) neurons’ intriguing behavior provides critical insights into the molecular mechanisms involved in excitotoxicity and fosters a critical re-evaluation of the role played by the metal in the modulation of age-related neurodegenerative diseases.

Aging is the primary risk factor for most neurodegenerative conditions, and evidence accumulated over the past 30 years has shown that brain aging is strongly associated with the production of Ca^2+^ dyshomeostasis and oxidative stress (Beckman and Ames, 1998; Alzheimer’s Association Calcium Hypothesis Workgroup, 2017). Zn^2+^ can concur in these processes.

### Zinc Dysregulation in AD

**FIGURE 2:**
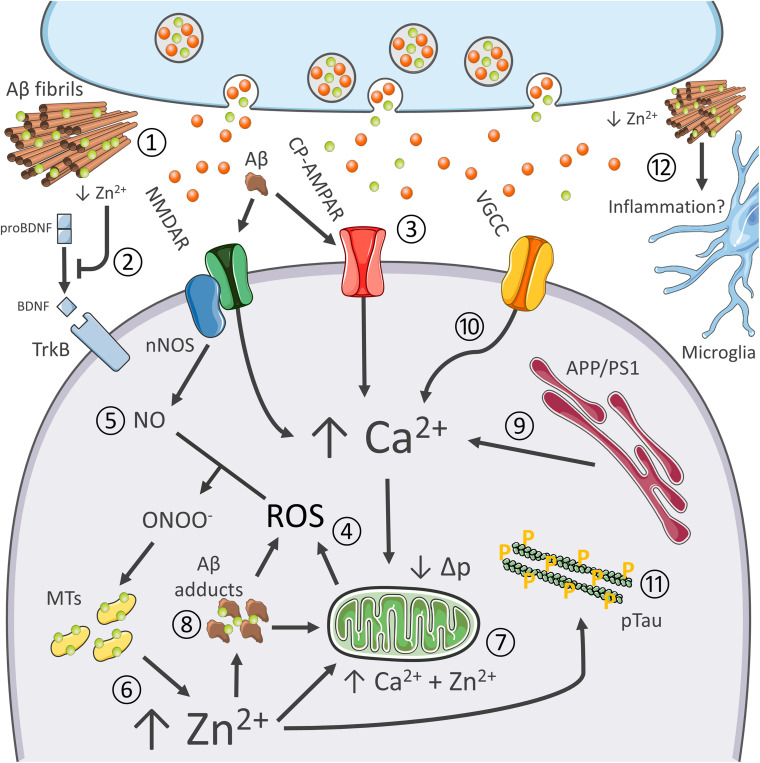
Synergistic contribution of Aβ and tau pathology, oxidative stress, excitotoxicity, Ca^2+^ dysregulation, and inflammation in AD. Role of Zn^2+^ in the process. The pictogram summarizes the synergistic interaction between AD-related molecular changes, and the cation dysregulation triggered by altered glutamatergic neurotransmission. Aberrant glutamatergic signaling represents a critical point of convergence of many of the molecular changes observed in AD. Aβ avidly sequesters synaptically released Zn^2+^ into senile plaques (1). Cation removal from the cleft negatively affects BDNF maturation and, therefore, impinges on neurotrophic signaling (2) as well as Zn^2+^-dependent NMDAR blockade. Aβ adducts can also directly activate NMDARs and CP-AMPARs, thereby further promoting the glutamatergic overdrive (3). NMDAR and CP-AMPAR overactivation promotes Ca^2+^ accumulation, increase the intracellular production of ROS (4), as well as the generation of nitric oxide (NO) from nNOS (5). ROS and RNS species are instrumental for Zn^2+^ release from MTs (6). Zn^2+^ build-up is potently neurotoxic as the cation, in synergy with Ca^2+^, further promotes mitochondrial dysfunction, ROS generation, and the release of apoptotic molecules (7). Zn^2+^ rises may also trigger intraneuronal Aβ aggregation (8). Aβ adducts may further contribute to mitochondrial impairment and generation of ROS. AD-related mutations on PS1 and APP and oxidative stress enhance Ca^2+^ dyshomeostasis by altering cation handling by the ER (9) and influx through VGCC (10). Ca^2+^ and Zn^2+^ rises, along with oxidative stress, also promote tau hyperphosphorylation (11). Finally, recent findings pinpoint at Zn^2+^ as a critical modulator of neuroinflammatory processes (12).

In an aging-dependent condition like AD, for example, Zn^2+^ affects β-amyloid (Aβ) metabolism and the resultant oxidative stress as well as promote tau pathology, the two hallmarks of the disease (Sensi et al., 2018; Figure 2). As for amyloid, Zn^2+^ is avidly sequestered by Aβ and found highly enriched in senile plaques of AD patients and AD transgenic mice (Bush, 2003; Granzotto et al., 2011). In that regard, a critical role is played by the metal released from presynaptic terminals. Crossing APP mutant mice with ZnT3 KO mice that lack pools of releasable Zn^2+^ dramatically decreases the Aβ burden (Lee et al., 2002). Of note, the ZnT3 KO mouse model has been proposed to phenocopy AD pathogenesis as the mice develop age-dependent cognitive deficits that overlap with the ones found in AD, thereby supporting the idea that the synaptic deficiency of the cation may participate in shaping disease progression (Deshpande et al., 2009; Adlard et al., 2010). Zn^2+^ released from presynaptic terminals by inhibiting AMPARs- and NMDARs is also critical for modulating excitatory neurotransmission (Paoletti et al., 2009; Figure 2). Disruption of this process has been associated with seizure-like activity in the hippocampus (Cole et al., 2000), a feature also observed in AD patients and preclinical AD models (Busche and Konnerth, 2015). In agreement, a substantial body of evidence also indicates that, in the early stages, AD is characterized by an aberrant glutamatergic activation (Busche and Konnerth, 2015), a process that leads to Ca^2+^ and Zn^2+^ dysregulation (Corona et al., 2011). In this context, the two cations can cooperate to promote the initial steps in the pathogenic cascade that generates the AD-related neuronal loss (Figure 2).

In the context of AD, multiple factors promote Ca^2+^ dysregulation. Altered glutamatergic neurotransmission represents a critical point of convergence of many of the molecular changes observed in AD, like vascular dysfunctions, metabolic deficits, and the aggregation of misfolded neurotoxic proteins (Sensi et al., 2018). AD-linked PS1 and APP mutation and accumulation of Aβ adducts can significantly interfere with both Ca^2+^ homeostasis and glutamatergic neurotransmission (Zhang et al., 2010; Kipanyula et al., 2012); Aβ-driven overactivation of glutamatergic signaling further exacerbate the process (Mattson et al., 1992; Figure 2). Of note, Aβ can also promote the endocytosis of NMDA-type glutamate receptors, a mechanism that, later on, may contribute to the synaptic failure observed in AD (Snyder et al., 2005). In parallel, Ca^2+^ dyshomeostasis alters APP processing and promotes neurofibrillary tangles (Figure 2).

Changes in cytosolic Ca^2+^ levels result in mitochondrial dysfunction, nitro/oxidative stress, and eventually neuronal death (Bezprozvanny and Mattson, 2008). Aβ further contributes to ROS’s toxic build-up elicited by Ca^2+^ dysregulation (Behl et al., 1994). In a self-feeding vicious cycle, oxidative stress can increase Ca^2+^ influx through VGCC (Todorovic and Jevtovic-Todorovic, 2014), Ca^2+^ release from intracellular stores through the redox-sensitive Ryanodine receptors (RyRs; Sanmartín et al., 2017), and impair mitochondrial Ca^2+^ buffering (Görlach et al., 2015).

The milieu triggered by Ca^2+^ dyshomeostasis and oxidative stress is therefore instrumental in setting in motion the neurotoxic activities of Zn^2+^ (Figure 2). The hypothesis that oxidative stress and Zn^2+^ dysregulation concur to promote damage in the aging AD brain is supported by evidence indicating age-dependent changes of molecules that control the brain metal homeostasis (Smith et al., 2006; Lyubartseva et al., 2010). For instance, the gene encoding expression for the neuronal isoform of MT3 is more abundant in aging hippocampal neurons (Giacconi et al., 2003; Scudiero et al., 2017) and AD brains (Hidalgo et al., 2006). While the abundance of MTs may reflect an endogenous protective response to a sub-chronic state of oxidative stress, the proteins can also offer increased availability of releasable Zn^2+^.

Although, still poorly investigated, Zn^2+^ dyshomeostasis may also participate in the functional and structural impairment of AD synapses. Evidence indicates that the metal modulates the assembly of critical components of the post-synaptic density (PSD; Grabrucker, 2014). The cation also coordinates the assembly of Shank scaffold proteins within the PSD (Tao-Cheng et al., 2016), complexes that, in turn, modulate synaptic stability and the recruitment of functional AMPARs (Ha et al., 2018). A key phenomenon as these receptors are critical modulators of long-term potentiation and depression processes that underlie memory and learning. Dietary Zn^2+^ deficiency, or cation sequestration by Aβ, negatively impinges the process, thereby potentially contributing to the synaptic dysfunctions observed in AD (Gong et al., 2009; Grabrucker et al., 2011).

Moreover, neuroinflammation is an emerging contributor factor in AD, and compelling evidence indicates that Zn^2+^ contributes to microglial activation (Kauppinen et al., 2008). Intriguingly, aspecific Zn^2+^-related transcriptomic changes have been recently described in xenotransplanted human microglia challenged with pro-inflammatory stimuli (Hasselmann et al., 2019).

Finally, as mentioned above, Zn^2+^ is also an essential component of MMPs (Page-McCaw et al., 2007). The enzymes, along with their activity on Aβ degradation, participate in the activation of BDNF (Hwang et al., 2005). Zn^2+^ can, therefore, modulate the neurotrophic axis and affect structural synaptic remodeling, two critical processes associated with learning and memory performances, and found impaired in AD (Weinstein et al., 2014). These findings support the notion of a causal link between Aβ dysmetabolism, Zn^2+^ dyshomeostasis, and the dysregulation of crucial brain signaling molecules (Figure 2).

### Zinc Dysregulation in Other Neurodegenerative Conditions

Zn^2+^ dysregulation may also contribute to HD, a condition characterized by the occurrence of a chronic glutamatergic overdrive, mitochondrial dysfunction, and oxidative stress and in which nNOS (+) neurons are spared (Ferrante et al., 1985; Leavitt et al., 2020). Although, still mostly unexplored, similar processes may take place in the motor neuron (MN) loss observed in ALS. MNs show selective expression of CP-AMPARs, thereby making this subpopulation particularly vulnerable to the glutamate-driven oxidative stress and Ca^2+^ dysregulation (Boillee et al., 2006; Weiss, 2011). The glutamate-Ca^2+^-Zn^2+^ cascade described here offers the rationale for exploring mechanisms involved in the degeneration of MN that occurs in ALS.

The selective vulnerability of dopaminergic neurons has been observed in PD, a phenomenon that, in analogy with HD and ALS, is associated with altered glutamatergic signaling. Furthermore, the neuronal loss in PD is also complemented by signs of mitochondrial dysfunctions and oxidative damage, processes that are also modulated by glutamate-driven Zn^2+^ dysregulation (Iovino et al., 2020).

Glutamate-driven Zn^2+^ dysregulation plays a significant role in ischemic and traumatic brain injury. Converging evidence accrued from preclinical stroke models indicates that the pharmacological chelation of the cation, as well as the reduction of mitochondrial Zn^2+^ uptake, prevent ischemic neuronal damage and the development of post-ischemic neurological sequelae (Koh et al., 1996; Frederickson et al., 2004; Zhao et al., 2018; Ji et al., 2019).

While a wealth of evidence has consolidated the notion that Zn^2+^ dysregulation plays an essential role in the glutamate-driven neuronal demise that occurs in stroke, brain trauma, and AD, less evidence has been gathered in other excitotoxic conditions like ALS, HD, or PD. This lack of information on the role of Zn^2+^ dyshomeostasis in these conditions constitutes the basis for a call to arms for the neurobiology community. This is becoming particularly relevant as a growing body of evidence is now demonstrating that the damaging processes set in motion in AD, PD, and ALS result from the synergistic activities of many polygenic, epigenetic, environmental, vascular, and metabolic factors, but most importantly make use of a final common neurodegenerative pathway (Sensi et al., 2018).

Furthermore, it is now clear that nosographic distinctions between different neurodegenerative conditions are becoming blurrier and blurrier. Most of the patients, if carefully investigated, exhibit a neuropathology mix characterized by different arrays of neurotoxic proteins like Aβ, tau, prion proteins (PrP), α-synuclein, and TAR DNA binding protein-43 (TDP-43) (Boyle et al., 2018; Karanth et al., 2020). This neuropathological mix is the rule and not the exception in AD and PD- and ALS-related neurodegeneration (Boyle et al., 2018; Karanth et al., 2020). Of great therapeutic and clinical importance, the molecular switches that act up or downstream of the “common neurodegenerative pathway” to turn the process toward the expression of specific clinical phenotypes are still not entirely known. We believe, and that is, in a nutshell, the main goal of this brief review paper, that further research is needed to fully disclose the role of Zn^2+^ in these processes and the generalizability of its damaging activities across several neurological conditions.

Targeting Zn^2+^ dysregulation appears, in fact, as a therapeutically exploitable venue (Lynes et al., 2007). Early studies employing clioquinol and its derivative PBT2, two Zn^2+^ (and copper) chelators, have shown promising effects in preclinical AD models (Cherny et al., 2001; Adlard et al., 2008), as well as in AD and HD clinical trials (Lannfelt et al., 2008; Faux et al., 2010; Angus et al., 2015). However, Zn^2+^ chelation therapy is not devoid of potential drawbacks. As indicated above, reducing brain Zn^2+^ levels could also negatively impinge on glutamate neurotransmission (Vergnano et al., 2014; Krall et al., 2020), neurotrophic signaling (Frazzini et al., 2018), and inflammation (Kauppinen et al., 2008; Olechnowicz et al., 2018). Thus, preclinical and clinical studies are needed to evaluate when, where, and how much Zn^2+^ dysregulation should be therapeutically addressed.

## Conclusion

In summary, by bridging Ca^2+^ dysregulation and oxidative stress, Zn^2+^ dyshomeostasis may represent a critical common event in many age-related neurodegenerative processes. Dissecting the complex *liaisones* between Ca^2+^, ROS, and Zn^2+,^ as well as deciphering their net contribution to specific disease-related pathological changes, may provide new clues for the development of novel therapeutic strategies. Finally, in the complex scenario of neurodegenerative conditions, considerable attention has been put on the selective vulnerability of specific neuronal subtypes (Saxena and Caroni, 2011; Surmeier et al., 2017; Fu et al., 2018). Therefore, the unique features of nNOS (+) neurons may lay the ground for the investigation of the molecular signature of “selective neuronal resilience.”

## Author Contributions

AG, LC, and SS conceived and designed the study and wrote the manuscript. All authors contributed to the article and approved the submitted version.

## Conflict of Interest

The authors declare that the research was conducted in the absence of any commercial or financial relationships that could be construed as a potential conflict of interest.
